# Bolstering Immunity through Pattern Recognition Receptors: A Unique Approach to Control Tuberculosis

**DOI:** 10.3389/fimmu.2017.00906

**Published:** 2017-08-02

**Authors:** Susanta Pahari, Gurpreet Kaur, Mohammad Aqdas, Shikha Negi, Deepyan Chatterjee, Hilal Bashir, Sanpreet Singh, Javed N. Agrewala

**Affiliations:** ^1^Immunology Laboratory, CSIR-Institute of Microbial Technology, Chandigarh, India

**Keywords:** tuberculosis, immunomodulation, innate cells, innate molecules, pattern recognition receptors

## Abstract

The global control of tuberculosis (TB) presents a continuous health challenge to mankind. Despite having effective drugs, TB still has a devastating impact on human health. Contributing reasons include the emergence of drug-resistant strains of *Mycobacterium tuberculosis (Mtb)*, the AIDS-pandemic, and the absence of effective vaccines against the disease. Indeed, alternative and effective methods of TB treatment and control are urgently needed. One such approach may be to more effectively engage the immune system; particularly the frontline pattern recognition receptor (PRR) systems of the host, which sense pathogen-associated molecular patterns (PAMPs) of *Mtb*. It is well known that 95% of individuals infected with *Mtb* in latent form remain healthy throughout their life. Therefore, we propose that clues can be found to control the remainder by successfully manipulating the innate immune mechanisms, particularly of nasal and mucosal cavities. This article highlights the importance of signaling through PRRs in restricting *Mtb* entry and subsequently preventing its infection. Furthermore, we discuss whether this unique therapy employing PRRs in combination with drugs can help in reducing the dose and duration of current TB regimen.

## Introduction

*Mycobacterium tuberculosis* (*Mtb*) is the causative agent of tuberculosis (TB). It remains a major health problem worldwide. It is responsible for over 10.4 million cases and 1.8 million deaths annually (1). About one-third of the global population is infected with *Mtb* but only 5–10% succumb to disease (2, 3). The failure of BCG vaccine to protect against TB, AIDS-pandemic, and the emergence of drug resistance of *Mtb* has further exacerbated the problem (4). The current lengthy regimen for TB treatment is full of complexity and inflicts patients with severe side-effects (5, 6). Hence, it is imperative to design novel and unique strategies that can overcome the problems associated with current treatment.

Host immunity successfully imparts optimum protection against the majority of pathogens (7–11). However, the success of *Mtb* to establish pathogenicity is due to its unique ability to skillfully tame and tune host immune responses and reside in the hostile environment, waiting for the right moment to take over the host immunity (11). Although our immune system sufficiently protects 90–95% of latently infected individuals from developing the disease, the remaining 5–10% of individuals are unable to restrict the growth of *Mtb*. T cells of adaptive immunity are known to play an important role in controlling the survival of the bacterium during latency, as well as in disease progression (12). Importantly, the role of innate immunity is considered more crucial than adaptive immunity since it acts as the first line of defense in combating the pathogen during the early phase of infection; much before adaptive immunity comes into operation. Adaptive immunity retains very high antigenic specificity through T cell receptors and B cell receptors. By contrast, innate immunity maintains specificity through pattern recognition receptors (PRRs) by recognizing conserved molecular structures that are expressed by a large variety of microbes known as “pathogen-associated molecular patterns” (PAMPs) (13). PRRs, such as toll-like receptors (TLRs), C-type lectin receptors (CLRs), NOD-like receptors (NLRs), dendritic cell (DC)-specific intercellular adhesion molecule-3-grabbing non-integrin (DC-SIGN), and mannose receptor are constitutively expressed on an array of cells that are devoted to perform functions not only for innate immunity but for adaptive immunity as well (14). Recently, the role of TLRs, CLRs, NLRs, and DC-SIGN have gained considerable attention following the observations that these molecules play a cardinal role not only in assisting the cells in capturing and internalization of the microbes but also in delivering the sequence of signaling events necessary for stimulating immunity and eliminating pathogens. Many PRR agonists are available commercially to study PRR-mediated activation of the immune response (15–18). Consequently, it may be quite important to utilize the agonists of PRRs as immunotherapeutic agents to control and eradicate *Mtb* infection and to minimize the chance of developing drug resistance.

## Role of Innate Immune Cells in Controlling the Propagation of *Mtb*

Our immune system is made up of a powerful combination of cells of innate and adaptive arms of immunity. In spite of the fact that innate immunity is the first line of defense against numerous invading pathogens, its importance has long been ignored due to the belief of its non-specific nature and inability to generate long-term protection (19). However, several studies have emerged during the last decades that could overcome this belief and establish the real importance of innate immunity. Innate immunity not only discriminates between the varieties of pathogens, including *Mtb*, but also contributes to sustained immunity (20–22). The importance of the cells of innate immunity can be accounted in individuals who are tuberculin-negative due to the absence of T cell immunity but are still protected against *Mtb* (23, 24). The sentinels of innate immunity include monocytes, macrophages, DCs, neutrophils, and natural killer (NK) cells. These cells contribute prominently in the eradication of *Mtb* by employing a variety of PRRs to recognize mycobacterium-specific PAMPs, such as carbohydrate and lipid moieties. Many PRRs directly elicit phagocytosis of *Mtb* and thereby stimulate secretion of cytokines, chemokines, and activation of the cascade of complement components. Complement components play a pivotal role in promoting the opsonization of pathogens.

*Mycobacterium tuberculosis* has a potential to virtually infiltrate every organ of the body, but lungs are the most preferred site for infection. The bacterium enters the nasal cavity and reaches lung *via* the respiratory tract. In the respiratory tract, neutrophils are the first cells to encounter *Mtb*. Neutrophils residing in our mucosa responds *via* neutrophil extracellular traps. After phagocytosing the *Mtb*, neutrophils alert the other cells of the immune system by releasing pro-inflammatory cytokines, free radicals, and chemokines (25). As a result, other components of respiratory mucosa, such as epithelial cells, connective tissues, macrophages, and DCs, get activated. Epithelial cells of mucosal lining have a potential to recognize the PAMPs of *Mtb* through their PRRs and subsequently, produce interferon (IFN)-γ, tumor necrosis factor (TNF)-α, and granzymes; which are the key contributors in the elimination of *Mtb* (26). Mucosal DCs present in the lung parenchyma and alveolar tracts are pivotal responders against *Mtb* (27). NK cells are the direct killers of infected macrophages and producers of major cytokines, such as type I IFNs and IFN-γ, which are essential for the activation of DCs and macrophages. Furthermore, even γδ T cells contribute in initiating the defense mechanism against *Mtb*. Moreover, γδ T cells act as antigen-presenting cells (APCs) to activate CD4 T cells, cross-present antigen to CD8 T cells, and produce IL-17 and IFN-γ in the lungs (28).

*Mycobacterium tuberculosis* can only reach the lungs upon successfully evading the innate immune response of the nose and upper respiratory tract. Henceforth, the nasal immunity is a strong checkpoint in controlling TB. The alveolar macrophages, which are highly professional phagocytic cells of innate immunity, are activated to engulf and destroy *Mtb*. In addition, DCs, neutrophils, and NK cells coordinate with each other in eliminating the bacteria. Correspondingly, APCs, such as DCs and macrophages, contribute in bridging the innate and adaptive immunity (20, 29). Macrophages and DCs possess PRRs that can sense PAMPs and their agonists, such as lipoarabinomannan, zymosan, CpG ODNs DNA, LPS, trehalose-6,6 dimycolate (TDM), TDB, curdlan, triacylated lipopeptide, MDP (muramyl dipeptide), and N-glycolyl MDP. Sensing of PAMPs by PRRs can induce and enhance the expression of MHC, co-stimulatory molecules, and adhesion molecules, as well as cause the release of many soluble mediators, such as cytokines, chemokines, and free radicals. In addition, PRRs induce the production of reactive oxygen intermediates, reactive nitrogen intermediates, and enhance apoptosis, autophagy, and inflammasome formation (30–35). Thus, optimal activation of various bactericidal mechanisms through immunomodulators can be an excellent approach to treat TB.

## Nasal and Oral Immune Defense: Frontline Barriers to Forbid the Entry of *Mtb*

The nasal cavity is the major site through which *Mtb* enters the lungs and establish infection. Therefore, strengthening nasal immunity is of pivotal importance. Thus, physical barriers of the nose and mucosal epithelium are important components of the nasal innate immunity that provide defense against *Mtb*. In this context, mucin produced by mucosal epithelium prevents the entry and attachment of the invading pathogen. Furthermore, the mucosa-associated lymphoid tissue employs various checkpoints against *Mtb*. Also, nasal immunity is highly enriched with phagocytic cells that are sufficiently endowed with the capability to prevent the entry as well as promote killing of *Mtb* by producing antimicrobial peptides, nitric oxide, lysozyme, defensins, mucins, cytokines, and chemokines (36). These phagocytic cells not only kill the pathogen at an early stage of infection but also process and present antigens to activate the cells of adaptive immunity (36). IgA produced by B cells contributes sufficiently against *Mtb* (37, 38). Hence, the cells of the innate immunity of nose coordinate with the adaptive immunity to control infections. Considering the extremely important role of nasal immunity, vaccines against polio, typhoid, cholera, rotavirus, and small pox have been exceptionally successful in eliminating these diseases by bolstering nasal immunity (39).

**Figure 1:**
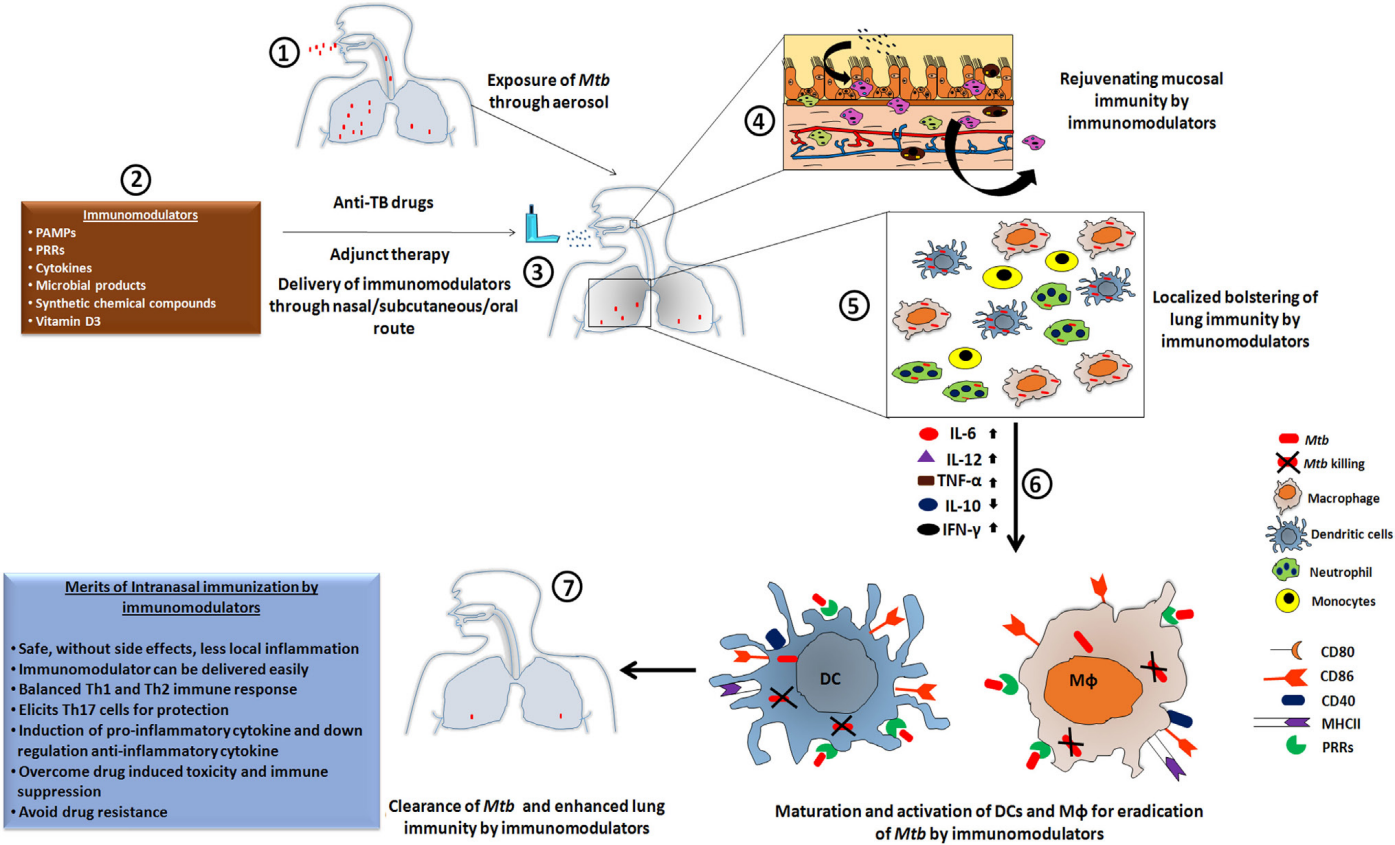
The heightened state of innate immunosurveillance is mediated through immunomodulators. [1] Here, we show a case in which a patient develops *Mtb* infection through the nasal route, leading to an influx of bacteria in the lung cavity to initiate the pathogenesis of tuberculosis (TB). [2,3] Such patients would be treated with a routine regimen of anti-TB drugs, but supplementation with immunomodulators could lead to a proactive exhibition of immune alertness by the local mucosal immune cells. [4] Subsequently, within the niche of the mucosal surface, the majority of bacteria would be eliminated; leaving only a few to escape the mucosal barrier and reach to the lung. [5] Adjuvant therapy has the ability to activate the downstream lung resident immune cells. These activated cells display a more potent phagocytotic activity, which is crucial for protection against the mycobacterium. [6] Subsequently, the upregulation of pro-inflammatory cytokines and the downregulation of their counterpart, due to the administered immunomodulator, results in the activation of co-stimulatory molecules that provides an important secondary signal. [7] Thus, the amalgamation of anti-TB drugs along with an immunomodulator as an adjuvant therapy leads to enhanced clearance of *Mtb* burden in a reduced time frame, lowers the chances of developing drug-resistant strains of *Mtb*, and reduces drug-induced toxicity and immunosuppression.

Another important compartment of the body that works as a checkpoint to control the infection is the oral cavity. It encompasses a well-compartmentalized network of cells that imparts a protective immunity and shields from invading pathogens (40). Recently, the role of commensals has been linked to elegantly contribute in boosting oral immunity (41–45). This highlights the importance of the cells of the oral cavity and commensals to subvert infection and protect against the pathogens. Importantly, bolstering the oral immunity through immunomodulators can be quite crucial in restricting *Mtb* pathogenicity. Potential targets for such therapy include the epithelial cells and resident mucosal DCs that have array of PRRs to trigger their activation (46). Since *Mtb* exploits both nasal and oral routes to enter the body and establish infection, there is a strong incentive to develop a successful vaccine against TB by adjuvanting resident immune cells of the nasal and oral cavity by targeting PRRs, thereby optimally boosting the immunity and, consequently eliminating invading mycobacterium at the entry point (Figure 1).

## Importance of Lung Immunity in Preventing *Mtb* Infection

**Figure 2:**
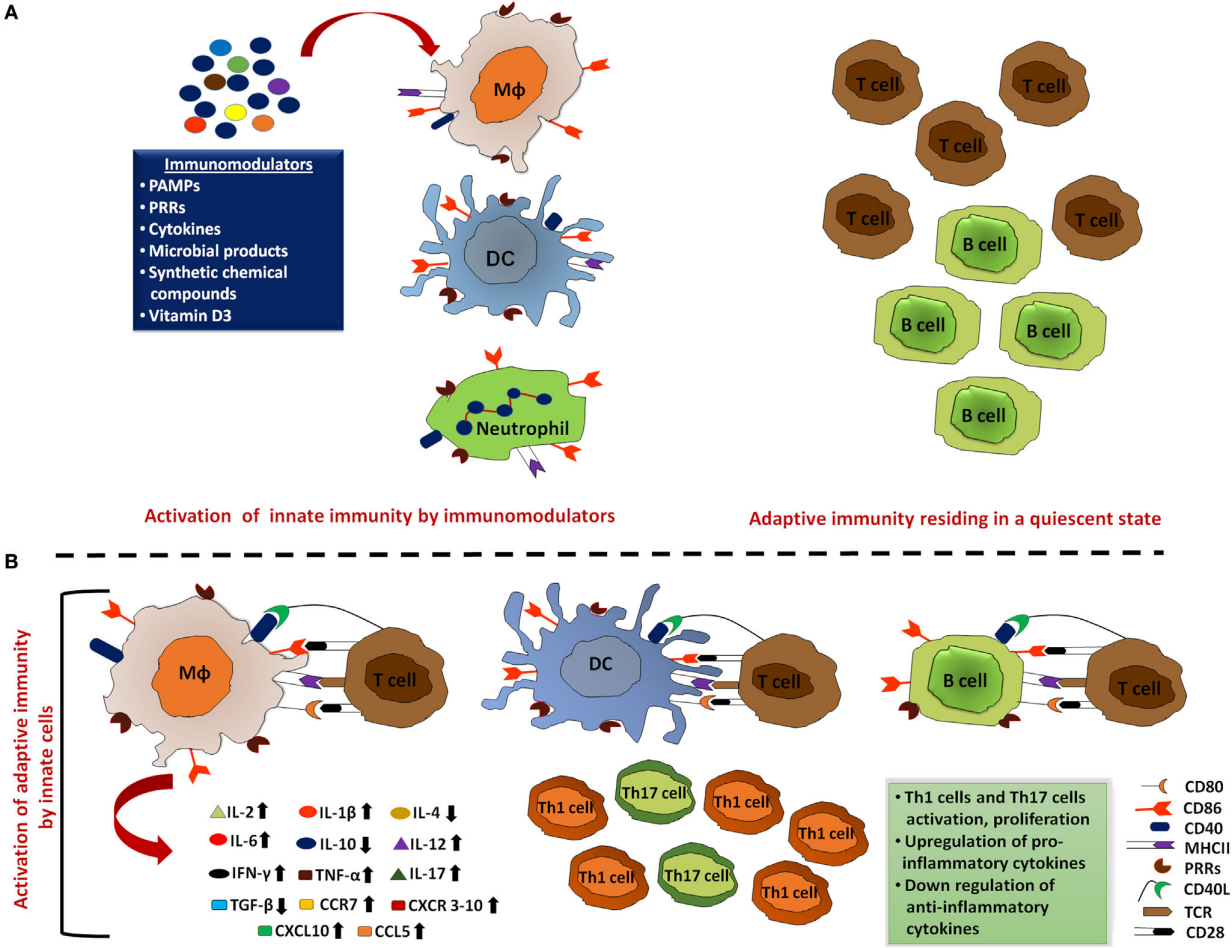
Activated innate immunity by immunomodulators meticulously clears the pathogens. **(A)** Innate cells that include macrophages, dendritic cells (DCs), and neutrophils act as the first line of defense to mount an immune response against the invading pathogens. Thus, aptly arming these cells by employing immunomodulators can ensure rapid activation of the innate cells and promote clearance of the majority of microbes during the initial encounter. In addition, immunomodulator-mediated upregulation of MHC, co-stimulatory molecules, and cytokines enhance the processing and presentation capability of these professional antigen-presenting cells (APCs). Such activation of innate cells effectively clears most of the pathogens without activating adaptive immune responses. **(B)** These immunologically activated innate cells also alert adaptive immune cells to ensure the elimination of remaining pathogens that escaped APC clearance. These activated T cells and B cells further induce pro-inflammatory cytokine production, inhibit the production of anti-inflammatory cytokines, and inhibit the activation and proliferation of Th1 and Th17 cells.

If *Mtb* evades nasal immunity and reaches lungs, both local immunity in the lungs and systemic immune responses are involved in combating the pathogen. Recently, many studies suggest a decisive role of immunomodulators in controlling infections and metabolic disorders (47–50). Unfortunately, not much has been explored in the case of TB. The lung is a highly vascularized organ and enriched with the cells of innate and adaptive immunity. Like nasal immunity, the cells of the innate immunity in lungs, such as alveolar macrophages, DCs, neutrophils, and NK cells, play a fundamental role in early detection of *Mtb* through PRRs and thereby alerting the cells of the immune system. Accordingly, the cells secrete an array of soluble mediators, such as free radicals, cytokines, chemokines, and elicit autophagy, apoptosis, and inflammasome formation to eradicate *Mtb* before it can effectively establish infection in the lung. Pulmonary collectins, hydrophilic surfactant proteins A and D (SP-A and SP-D) are known to regulate pulmonary host defense and inflammation. SP-A and SP-D are produced predominantly by type II alveolar cells. It has been reported that SP-A and SP-D play pivotal role in promoting lung innate immunity (51). SP-A and SP-D are known to directly interact with macrophages and enhance their phagocytic potential against pathogens, such as *Streptococcus pneumoniae* and *Mtb* by upregulating the cell surface localization of phagocytic receptors (52). Interestingly, majority of tuberculin-negative individuals remain protected throughout their lifetime against *Mtb* (1, 23, 24). Thus, signifying that not only adaptive immunity plays the crucial role but innate immunity can efficiently contribute in controlling the disease (Figure 2).

Therefore, it may be crucial to consider both adaptive and innate immunity in enhancing the nasal immune responses to prevent *Mtb* reaching the lung. In connection with this, understanding the sequence of signaling events operating through PRRs to optimally activate immunity against *Mtb* can be decisive in developing or designing adjuvants that can substantially supplement vaccines and immunotherapies.

## Targeting PRR-Associated Molecules to Regulate Innate Immunity

### Toll-Like Receptors

Toll-like receptors are the most extensively studied class of PRRs. They are one of the components of the immune system to first encounter pathogens. Structurally, TLRs are type I transmembrane proteins that are composed of an ectodomain, a transmembrane region, and cytosolic Toll-IL-1 receptor (TIR) domain that trigger downstream signaling pathways. TLRs recognize a wide array of extracellular or intracellular PAMPs (53). So far, around 10–12 functional TLRs have been identified in both mice and humans. Each TLR detects discrete PAMPs derived from distinct classes of pathogens, including bacteria, viruses, fungi, and parasites. These include lipoproteins (TLR-1, TLR-2, and TLR-6); double-stranded (ds) RNA (TLR-3); LPS (TLR-4); flagellin (TLR-5); single-stranded (ss) RNA (TLR-7 and TLR-8); and CpG ODNs DNA (TLR-9) (53). Upon PAMP recognition, TLRs recruit a specific set of adaptor molecules, which bind to TIR domains, such as MyD88 (myeloid differentiation primary response gene 88) and TRIF (TIR-domain-containing adapter-inducing IFN-β), and initiate downstream signaling events, such as secretion of inflammatory cytokines, chemokines, type I IFNs, and antimicrobial peptides (21). Ultimately, these responses initiate a cascade of sequestered processes, such as neutrophils recruitment, macrophage activation, and induction of IFN-stimulated genes, thereby resulting in the direct killing of pathogens (19, 21). *Mtb* is known to mediate cellular activation through TLR-2 and TLR-4. Mycobacterial lipoproteins, soluble heat-stable, and protease-resistant factors can elicit the immune response through TLR-2 signaling, leading to apoptosis and killing of *Mtb*. By contrast, heat-sensitive membrane-associated factors operate *via* TLR-2 and TLR-4 pathways (54–56). Also, it has been shown that TLR-4 mutant C3H/HeJ mice have an increased susceptibility to *Mtb* infection due to reduced macrophage recruitment and diminished pro-inflammatory cytokine responses (57). Our group has recently demonstrated that signaling through TLR-2 with its ligand Pam2Cys can rescue Th1 cells from undergoing exhaustion during chronic *Mtb* infection (17, 58). Furthermore, the therapeutic potential of self-adjuvanted chimeric vaccine comprising TLR-2 agonist-Pam2Cys and MHC class II binding promiscuous peptide derived from 16 kDa antigen of *Mtb* showed a significant decline in *Mtb* burden by expanding the pool of effector as well as memory Th1 cells and Th17 cells (59). It has been reported that TLR-9 present on the APCs recognizes *Mtb*-derived unmethylated CpG, subsequently triggering a potent Th1 response (60), thus signifying the role of TLRs in controlling *Mtb* infection.

### C-Type Lectin Receptors

C-type lectin receptors belong to a large superfamily of proteins containing one or more C-type lectin domains (61, 62). In vertebrates, CLR family is diversified into 17 subclasses. CLRs are expressed on DCs, macrophages, and NK cells that are involved in the recognition of various pathogens including *Mtb* (63–65). Some CLRs exist in a soluble form such as mannose-binding lectin (MBL), while others as transmembrane proteins such as Dectin-1. MBL is among the best-known CLRs that is found in the serum, activates the complement system, and binds to a wide range of carbohydrate motifs present on bacteria, viruses, protozoa, and fungi (66). Certain CLRs can recognize oxidized lipids and protein ligands expressed by apoptotic or necrotic cells (67–69). CLRs contain one or more extracellular carbohydrate recognition domains (CRDs), some of which contain Ca^2+^ binding sites. Certain amino acids in the CRD determine the carbohydrate specificity of the CLRs. It has been reported that CLRs expressing the amino acid motif EPN (Glu-Pro-Asn) in the CRD are specific to mannose-based ligands, whereas galactose-specific CLRs often express QPD (Gln-Pro-Asp) (70). The ligand specificity and the immunological signaling vary between different CLRs. For example, Dectin-1 recognizes certain β-glucan, thus initiating pro-inflammatory signaling. In addition, Clec9a binds to filamentous actin (F-actin) expressed in the necrotic cells of the body and induces cross-presentation of antigens to APCs (62, 69, 71).

Dectin (Dectin-1 and Dectin-2) represents the archetype of non-TLRs, falling in type II transmembrane proteins. Dectin comprises extracellular CRD connected by stalk to transmembrane domain and cytosolic ITAM (immunoreceptor tyrosine activation motif) (72). Dectin is a pathogen recognition CLR expressed on macrophages, neutrophils, DCs, Langerhans cells, and the certain subset of T cells. Cells expressing Dectin recognize fungal wall-derived β-glucans and can confer protection against various infections (73). Furthermore, signaling through Dectin has been suggested to play an important role in host immunity against *Mtb*. Alpha-glucan of *Mtb* is known to bind Dectin-1 receptor and promote the uptake of *Mtb* (73, 74). In addition, *Mtb* induces the expression of Dectin-1 in the epithelial cells through TLR-2 dependent signaling; leading to the production of free radicals and pro-inflammatory cytokines, such as TNF-α, IL-6, and CXCL8 (75). *Mtb*-infected Dectin^−/−^ mice showed the decline in IL-12p40 yield by DCs and, therefore, reduced immunity against *Mtb*. Consequently, these studies highlight the importance of Dectin-1 in imparting immunity against the bacterium (76). Many studies suggest that binding of Man-LAM (mannose lipoarabinomannan) to Dectin-2 elicits the production of pro- and anti-inflammatory cytokines (77, 78). Furthermore, it is suggested that *Mtb*–Dectin interaction activates Syk/CARD9 signaling pathway and delivers protective response (79). Upon *Mtb* infection, Dectin-1 is upregulated on alveolar macrophages, leading to respiratory burst and production of pro-inflammatory cytokines, such as IL-6, IL-12, and IL-17 (75, 80). Furthermore, Dectin-1 in conjunction with TLR-2 is known to play an important role in DC maturation upon infection with *Mtb* (81). It enhances reactive oxygen species production, leading to activation of various MAPKs and Src kinases and pro-inflammatory response against *Mtb* (75, 82). By contrast, *Mtb* also employs its virulent factors, such as TDM, phosphatidylinositol, and Man-LAM, to bind to CLR and infect host cells (83). Man-LAM can suppress phagosome–lysosome fusion and help in the intracellular survival of *Mtb*.

Activation of CLR induces signal transduction and gene transcription promotes phagocytosis and enhances the potential of DCs to stimulate T cells (64). The recognition of PAMP by Dectin-1, Dectin-2, and Mincle (macrophage-inducible C-type lectin) initiates kinase Syk signaling; eventually leading to NF-κB induced transcription (64, 66, 84, 85). Mincle and Dectin-2 recognize the gamma-chain of Fc receptor as an adaptor molecule to trigger activation of cells (84–87). DC-SIGN recognizes fucose or mannose moieties on numerous pathogens, such as *Mtb, Candida albicans*, measles virus, and HIV-1, which triggers Raf-1-dependent signaling pathway and initiates TLR-induced NF-κB activation (88, 89). Indeed, more in-depth studies are required to understand the interaction between CLRs and *Mtb* to decipher their role in therapeutic intervention.

### NOD-Like Receptors

Recently, a family of PRRs known as nucleotide binding and oligomerization domain NLRs has gained considerable impetus following their substantial contribution in host–pathogen interaction. In the case of humans, 22 NOD molecules are reported. Their basic structure includes a central NACHT domain and a carboxy-terminal leucine-rich repeat region (90–92). The NLR proteins are cytosolic sensors for the bacterial cell wall component such as peptidoglycan (93, 94). One subfamily of NLRs includes NOD-1 and NOD-2, which have an amino-terminal caspase recruitment domain (CARD), essential to trigger NF-κB signaling. This activation leads to the enhanced release of pro-inflammatory cytokines (IL-1β, IL-6, TNF, and IL-8), chemokines, nitric oxide, and antimicrobial peptides (β-defensin 2). This leads to an increased expression of co-stimulatory and adhesion molecules on mononuclear cells (95–100). NOD-2 along with adaptor protein CARD9, augment MAPK, JNK, and p38 signaling pathways (101). Recently, NOD-2 has been involved in the regulation of IRF3 signaling and type I IFN production in response to the ssRNA of respiratory syncytial virus (102). The importance of NLRs family is reflected by the fact that mutations in these genes can lead to many diseases. The role of NLRs has been extensively studied in several bacterial and viral infections, inflammatory bowel disease, Crohn’s disease, granulomatous inflammatory disorders, such as early onset of sarcoidosis, Blau syndrome, so on (103–105). Nonetheless, their role in TB remains inconclusive. *Mtb*-infected NOD-2^−/−^ mice showed impaired cytokine production by macrophages and DCs (106). Furthermore, these animals exhibited higher bacterial burden in the lungs and reduced survival due to impaired T cell function (107), suggesting that NOD-2 is crucial in the generation of *Mtb*-reactive T cells. It may be an interesting line of investigation to study the therapeutic role of NOD-2 in bolstering T cells activity during *Mtb* infection. NOD-2 is known to act synergistically with TLR-2/TLR-4 to promote the release of inflammatory cytokines during *Mtb* infection (108, 109). Recently, our group has shown that activating DCs through NOD-2 and TLR-4 successfully restricted the intracellular survival of *Mtb* (16). Such DCs acquired enhanced ability to present antigen to CD4 T cells and CD8 T cells. These T cells exhibited improved release of IFN-γ and TNF-α cytokines that play a cardinal role in protection against *Mtb*. Furthermore, sustenance and expansion in the pool of memory T cells was noticed (110). Interestingly, adjunct therapy to treat *Mtb*-infected mice using the ligand of NOD-2/TLR-4 with anti-TB regimen improved the efficacy of drugs by reducing the dose, without compromising their potency to kill *Mtb* (16).

## Cytokines Secreted by the Cells of Innate Immunity Act as Immunomodulators Against *Mtb*

Cytokines are low molecular weight glycoproteins produced by various cells of the innate and adaptive immunity. They play a paramount role in the activation and differentiation of immune cells; maintain a fine balance of homeostasis and communication network between the cells of immune system. Any disturbance in these events may instigate autoimmunity, improper protection from infectious diseases, and aberrant growth of tumors (111). Immunity can be modulated by an array of cytokines, including IL-1β, IL-2, IL-4, IL-6, IL-10, IL-12, IL-17, IFN-β, IFN-γ, TNF-α, and TGF-β, etc. (111). Currently, immunomodulatory role of cytokines is being exploited in various diseases. It has been suggested that IL-2- and IL-15-activated NK cells acquire potent anti-cancer activity (112, 113). Other examples include the role of α-IFNs in modulating the activity of vaccine against hepatitis B virus and pegylated IFNs with ribavirin for the treatment of hepatitis C virus (111). Recently, a synthetic form of IFN-α2b known as Infergen showed a potent immunomodulatory activity against *Mtb*, by the activation of macrophages, induction of autophagy and inhibition of the growth of *Mtb* (114). It is well known that cytokines can aptly improve the efficacy of vaccines. Co-administration of memory enhancing cytokines IL-7 and IL-15 substantially augmented the efficacy of BCG by eliciting enduring memory T cells and protection against *Mtb* (115). Similarly, IL-1, IL-6, and TNF-α bolstered BCG potency by generating long-lasting protective memory T cell response (116). Thus, it would be quite interesting to develop a recombinant BCG expressing memory enhancing cytokines. This strategy would improve the efficacy of BCG in protecting not only childhood but also the adult manifestation of TB. It is worth to mention here that BCG protects only children against TB but its efficacy wanes with the age (117).

## Microbial Products

In 1885, Louis Pasteur hypothesized that microbes possess immunogenic components. These microbial products include several molecules that can be sensed by the PRRs of the host to alert the cells of the immune system (118). Triggering of DCs and macrophages by the microbial PAMPs releases numerous cytokines and chemokines. MDP of *Mtb* is endowed with adjuvant properties since it can bind to NOD-2 of host cells and boosts their function (16, 109, 119). Furthermore, monophosphoryl lipid A (MLA), a lipopolysaccharide component of Gram-negative bacteria cell wall is an agonist of TLR-4. MLA has been established to possess adjuvant activity and induces the immune response to heterologous proteins, making it an integral part of vaccines and immunotherapeutics (120, 121). The unmethylated CpG of *Mtb* recognized by TLR-9 activates Th1 cells preferentially (60, 122). Likewise, glucans, zymosan, lentinans, and aminated poly glucose of yeast activate macrophages and provide optimum antimicrobial immunity to the host. The bacterial lipoproteins act as a potent adjuvant by delivering danger signal through TLR-1/2, which stimulates the production of IL-1, IL-6, TNF-α, and NO by monocytes and macrophages, which ultimately strengthen the immunity (123). Many subunit vaccines against intracellular pathogens fail to reach clinical trials due to the non-availability of adjuvants that can be approved for human use. Microbial-derived adjuvants efficiently evoke the cell-mediated immune response (124–126). In addition, they are highly stable and less toxic (123), making them suitable for human use; especially for vaccines against TB and HIV.

## Synthetic Chemical Compounds

A wide array of microbial components is known to induce immunomodulatory activities (127). In past, these agents have been successfully used in designing or modifying potent vaccine candidates and drugs against pathogens and inflammatory responses (128). These synthetic compounds include mono- and multivalent galabiose derivatives, glycol-dendrimers, galactose-dendrimers, glycopeptide-dendrimers, and multivalent glycosylated fullerenes, which elicits a potent immunogenic activity (129). Microbes also possess certain essential cell surface carbohydrates, glycoconjugates, and oligosaccharides; which fundamentally help in the cell growth, proliferation, and cell–cell interactions. These molecules are well recognized by parenchymal cells, endothelial cells, and immune cells as they express specific PRRs to recognize microbial components. Chemically synthesized analogs of these microbial components, such as MLA, have been identified as potent immunomodulatory compounds that can enhance immunity and act as adjuvant for several vaccines (121).

## Role of Vitamin D3 as a Potential Immunomodulator in TB Treatment

1,25-Dihydroxyvitamin D3 (VD3) has been reported for inducing host resistance against TB. VD3 has been used as a mode of treatment during the early pre-antibiotic age. VD3 acts through its receptor and its genetic polymorphisms have been linked with susceptibility or resistance to TB. Recent studies demonstrate that VD3 boosts innate immunity by improving the expression of various antimicrobial peptides and induction of autophagy that ultimately restricts the intracellular growth of *Mtb*. Furthermore, VD3 has been involved in coordination with various pro-inflammatory cytokines, such as IL-15, IL-32, and IFN-γ, to control TB (130, 131). Various clinical trials have assessed VD3 as a probable candidate for adjunct therapy to treat TB (132, 133). Henceforth, a combinatorial therapy of VD3 and anti-TB drugs may be a novel therapeutic strategy to treat patients suffering from TB.

## Inhibition of Immune Checkpoints

Recently, antibody therapy to block the function of checkpoint inhibitors, such as the programmed cell death-1 (PD-1) and cytotoxic T-lymphocyte-associated antigen 4 (CTLA-4), has gained substantial importance in overcoming the suppression induced by these molecules. Some clinical studies indicate a potential contribution of the PD-1/PD-L1 pathway in the progression of TB pathogenesis. PD-L1 is constitutively expressed on the macrophages of patients infected with *Mtb* (134). Blockade of PD-L1 with its monoclonal antibodies enhances IFN-γ production by T cells and help to control the growth of *Mtb* in pulmonary TB patients (134, 135). Furthermore, PD-1 expressed on the surface of regulatory T cells (Tregs), neutrophils, and NKT cells control the inflammatory response, avoiding damage done to patient tissues (136–139). It has been demonstrated that CTLA-4 is equally expressed by effector T cells and Tregs isolated from the blood of active TB patients, which may be important in maintaining the optimum immune response (136, 140, 141).

Another immune checkpoint, lymphocyte-activation gene-3 (LAG-3), is commonly expressed by Tregs cells. Blocking of LAG-3 modulates CD4 T cells response and avoids T cell exhaustion (142). Recently, LAG-3 has been shown to be highly expressed in the lungs of patients suffering from active TB but not in latent infection (143). In addition, T cell immunoglobulin and mucin domain-3 (TIM-3) bind to galectin 9 (Gal9) on the surface of APCs. Blocking of TIM-3 prevents apoptosis of *Mtb*-reactive effector T cells (144). *In vitro* TIM-3 blockade in co-culture experiments with *Mtb*-infected macrophages and T cells isolated from TB patients fostered bacterial killing by enhancing IL-1β secretion by macrophages along with IFN-γ release by T cells (145, 146). Neutralization of the activity of TIM-3 and PD-1 by their respective antibodies resulted in enhanced T cells response and reduction of *Mtb* burden in the macrophages obtained from HIV–TB patients (146). These studies suggest that blocking of immuno-checkpoint molecules significantly improve the immunity of the host. It may be an interesting line of investigation to treat TB patients with drugs while boosting their immunity by blocking the function of immune checkpoint molecules by their respective antibodies.

## Immunomodulation through PRRs: An Approach to Overcome Drug Resistance in *Mtb*

Recent reports from WHO reveals that antibiotic treatment will not be successful beyond 2020 due to the emergence of totally drug-resistant strains of *Mtb* (147–149). Drug-resistant strains of *Mtb* have evolved to evade antibiotic treatment by several mechanisms, including efflux pumps, mutations of antibiotic target proteins, and development of enzymes that degrade the active moieties of an antibiotic (150–157). Lengthy drug treatment is required to clear the *Mtb* infection. The excessive and prolonged use of anti-TB drugs is a major contributor in promoting the development of drug resistance. Stimulating the host cells through PRRs can overcome this problem. It is now a well-established fact that signaling through PRRs can optimally activate the cells of both innate and adaptive immunity (15–18). Signaling delivered through PRRs can augment APC capacity to sense and phagocytose *Mtb* followed by processing and presentation of its antigens to T cells. This process is essential for the clearance of infection (15). A combinatorial therapy to stimulate the immune system by immunomodulators and drugs to kill *Mtb* in a concerted fashion can substantially control the development of drug resistance and will help to reduce the dose and duration of treatment. Furthermore, it will overcome the toxicity and immunosuppression that are associated with current regimen.

**Figure 3:**
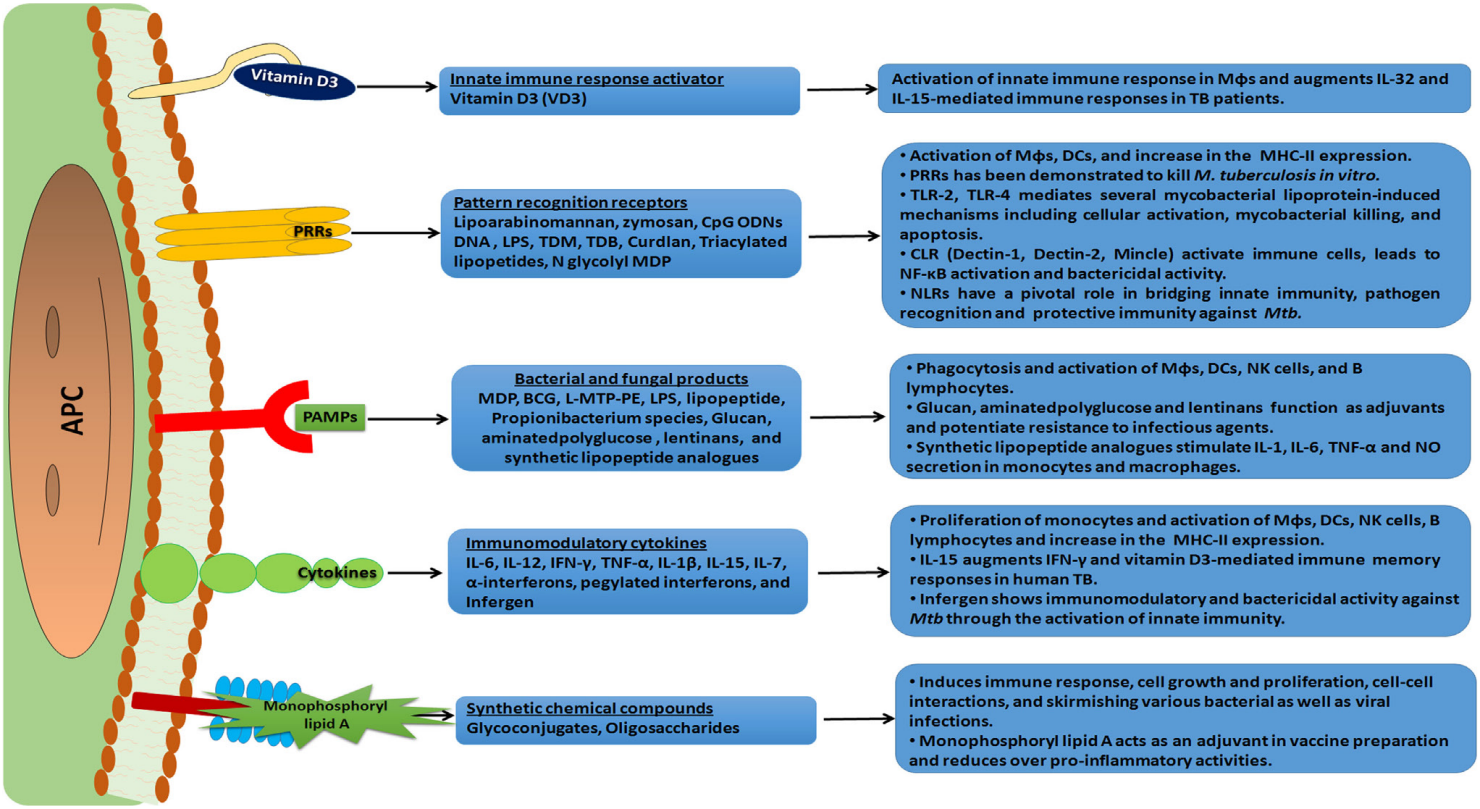
An overview of immunomodulation and mode of action of various immunomodulators. An array of immunomodulators that can be effectively used as a host-directed therapy against the invading pathogen is shown. These molecules have receptors on antigen-presenting cells (APCs) and trigger a cascade of intracellular signal transduction events. This subsequently leads to a heightened state of immune alertness, ensuring an effective quarantine of the pathogen and the elimination of a significant number of pathogens. Such a therapy can reduce the dose of anti-tuberculosis (TB) drugs and minimize the chances of developing drug resistant strains of *Mtb*.

The challenge still remains to identify the appropriate agonists of PRRs that can deliver optimum response against *Mtb* (Figure 3). This may require extensive investigation to select the most potent PRRs from different TLRs (TLR-2, TLR-4, and TLR-9), NLRs (NOD-1, NOD-2), and CLRs (Mincle, Dectin-1, and Dectin-2) (15, 30–35, 158). In this context, an elegant study conducted in the experimental model of TB has indicated that adjunct therapy involving agonist of NOD-2 and TLR-4 can reduce the dose of anti-TB drugs (16). It will be an interesting line of future investigation to study the potency of NOD-2 and TLR-4 in TB patients to shorten the current treatment.

## Precautions While Modulating the Immune System

In this article, several advantages of stimulating the immune system through molecules of innate immunity have been discussed. However, side-effects that can incur due to hyperactivation of the immune systems, such as inflammatory responses and tissue damage, cannot be totally denied. Such therapies cannot be given to individuals suffering from neurodegenerative diseases, arthritis, asthma, inflammatory bowel disease, or lung fibrosis (159). Furthermore, cryptic antigens released by hyperactivation of the immune system may break immune tolerance, leading to undesired activation and proliferation of autoreactive T cells and B cells, eventually culminating into autoimmune diseases (160). Furthermore, the excessive release of pro-inflammatory cytokines and free radicals will damage host tissues, as observed in the case of sepsis (148, 158, 161–166). Similarly, over stimulation can have adverse side-effects on individuals suffering from allergies, autoimmune diseases, and inflammatory disorders. It is crucial to take into consideration immune status of TB patients while treating them with immunomodulators for controlling *Mtb* infection. Consequently, understanding the history of the patient will be important before practicing such therapies. Therefore, to deliver maximum benefit, immunomodulators should be used in a form of a personalized medicine.

The inflammatory responses generated through immunomodulation can be well controlled by careful titration of the dose of an immunomodulator. One of the possibility is to administer the immunomodulators through nasal route at the site of infection i.e., lungs during TB infection. This will elicit local immunity that is crucial to combat *Mtb* without provoking the undesirable activation of systemic immunity. In this context, a detailed and broad spectrum pharmacological study to decipher any adverse effect of immunomodulatory therapy with other drugs is of paramount importance. Importantly, a particular immunomodulator may not be potent against all pathogens. Therefore, it requires a thorough examination of its efficacy against certain pathogens. Despite these limitations, immunomodulation can be quite effective because it targets the host rather than the pathogen, thus avoiding the chance of evolving drug resistance in microbes. In addition, boosting innate immunity offers an advantage of providing protection against an array of pathogens. Furthermore, in combination with the standard drugs, it can minimize the side-effects; and reduce the dose and duration of lengthy TB regime.

## Therapeutic Intervention

Despite of the fact that potent drugs are available to treat TB, the disease continues to devastate human lives. Furthermore, lengthy drug regime provides sufficient time and opportunity for the bacteria to develop drug resistance. Furthermore, the situation is complicated due to AIDS-pandemic and non-compliance of BCG vaccine to protect from the disease (117, 167). Therefore, there is an urgent need and challenge for the scientific community to devise and develop alternative means to treat TB patients. In this regard, one of the promising and novel approaches can be “host-directed therapy,” by exploiting the potential of PRRs to elicit anti-TB immunity. Such a strategy can be explored as an adjunct therapy with the drugs to reduce the dose and duration of treatment. In addition, this approach will have added advantage in controlling the emergence of drug resistance in *Mtb* by eliciting host immunity, thereby increasing the potency of drugs. Additionally, in the age of “one world one health one medicine” theory, the persuasive utilization of immunomodulators can be considered as an exclusive and alternative means to treat patients suffering from many infections.

## Author Contributions

The designing of theme and concept were done by JA and SP. The figures and manuscript were prepared and written by SP, GK, MA, SN, DC, HB, SS, and JA.

## Conflict of Interest Statement

The authors declare that the research was conducted in the absence of any commercial or financial relationships that could be construed as a potential conflict of interest.
